# Treatment fractionation for stereotactic radiotherapy of lung tumours: a modelling study of the influence of chronic and acute hypoxia on tumour control probability

**DOI:** 10.1186/1748-717X-9-149

**Published:** 2014-06-30

**Authors:** Emely Lindblom, Laura Antonovic, Alexandru Dasu, Ingmar Lax, Peter Wersäll, Iuliana Toma-Dasu

**Affiliations:** 1 Medical Radiation Physics, Department of Physics, Stockholm University, Stockholm, Sweden; 2 Department of Radiation Physics and Department of Medical and Health Sciences, Linköping University, Linköping, Sweden; 3 Department of Radiation Physics, Karolinska University Hospital, Stockholm, Sweden; 4 Department of Oncology, Karolinska University Hospital, Stockholm, Sweden; 5 Medical Radiation Physics, Department of Oncology and Pathology, Karolinska Institutet, Stockholm, Sweden

**Keywords:** Hypoxia, Hypofractionation, SBRT, NSCLC

## Abstract

**Background:**

Stereotactic body radiotherapy (SBRT) for non-small-cell lung cancer (NSCLC) has led to promising local control and overall survival for fractionation schemes with increasingly high fractional doses. A point has however been reached where the number of fractions used might be too low to allow efficient local inter-fraction reoxygenation of the hypoxic cells residing in the tumour. It was therefore the purpose of this study to investigate the impact of hypoxia and extreme hypofractionation on the tumour control probability (*TCP*) from SBRT.

**Methods:**

A three-dimensional model of tumour oxygenation able to simulate oxygenation changes on the microscale was used. The *TCP* was determined for clinically relevant SBRT fractionation schedules of 1, 3 and 5 fractions assuming either static tumour oxygenation or that the oxygenation changes locally between fractions due to fast reoxygenation of acute hypoxia without an overall reduction in chronic hypoxia.

**Results:**

For the schedules applying three or five fractions the doses required to achieve satisfying levels of *TCP* were considerably lower when local oxygenation changes were assumed compared to the case of static oxygenation; a decrease in *D*_50_ of 17.7 Gy was observed for a five-fractions schedule applied to a 20% hypoxic tumour when fast reoxygenation was modelled. Assuming local oxygenation changes, the total doses required for a tumor control probability of 50% were of similar size for one, three and five fractions.

**Conclusions:**

Although attractive from a practical point of view, extreme hypofractionation using just one single fraction may result in impaired local control of hypoxic tumours, as it eliminates the possibility for any kind of reoxygenation.

## Background

The use of stereotactic body radiation therapy (SBRT) has continuously grown and proven highly successful in the treatment of lung cancer [1-9] since the first treatments of extra-cranial malignancies employing few high-dose fractions performed by Blomgren and Lax in the 1990s [1,2]. As treatments delivered in fewer fractions are more advantageous from both economical and practical points of view, there is a tendency towards extreme hypofractionation in SBRT. The high precision allowed by the use of a stereotactic frame to fixate the patient or, more recently, image-guided frameless techniques has enabled an escalation of the fractional dose. However, the impact of extreme hypofractionation on the treatment outcome must also be evaluated from a radiobiological point of view as such schedules may pose a challenge to the radiobiological rationale behind fractionation summarized by the so-called 5 R’s of radiobiology. A reduced number of fractions implies a shorter treatment time, but also requires a higher dose per fraction to achieve the same effect. Therefore, the impact of redistribution and repopulation can be neglected as the high doses will most likely cause cell cycle arrest [10] and accelerated repopulation does not usually occur until after about four weeks of conventional radiotherapy [11]. Furthermore, due to the high dose rates of today’s modern accelerators, repair during delivery will also be negligible [12].

Particular attention might be required for the hypoxic cells that are more likely to survive irradiation due to their increased radioresistance compared to well-oxygenated cells [13]. For conventional fractionation, reoxygenation of hypoxic tumour cells during therapy is considered a crucial process as some of the hypoxic cells are assumed to become oxic between fractions and thus radiosensitized at the next fraction [14,15]. Although no improvement in the global oxygenation status through tumour shrinkage could be expected during a short SBRT treatment course [13], experimentally observed inter-fraction local changes in oxygenation might benefit the treatment outcome [16]. The reduced possibility of these local changes implied by a considerably reduced number of fractions might however compromise the treatment outcome for patients with hypoxic tumours. The oxygenation, together with the related radiosensitivity of a tissue, should thus be considered in evaluating the impact of extreme hypofractionation. Previous studies on this topic by Ruggieri *et al.*[17] and Carlson *et al.*[18] led to seemingly conflicting conclusions on how SBRT-like treatment schemes impact upon the treatment outcome in hypoxic tumours*.* The present study adds to the previous work and aims to bring further arguments that may clarify the impact of hypoxia on tumour control probability (*TCP*) when the dose is delivered in very few, large fractions.

## Method and materials

### Calculation of surviving fraction

The study was performed on voxelized three-dimensional models simulating tumours with a diameter of 20 mm and heterogeneous oxygenations as previously described by Dasu *et al.* (2003, 2005) and Toma-Dasu *et al.* (2009) [19-21]. Cell response to the treatment was calculated using two different cell survival models, the linear-quadratic (LQ) model [22] and the universal survival curve (USC) model [23]. There is an on-going debate on whether the well-established LQ model overestimates the cell-kill for the high doses per fraction typically employed in SBRT [24]. The universal survival curve model, which is an empirical joining of the LQ model at low doses and the single-hit multi-target (SHMT) model at higher doses causing an exponential fall-off in survival as opposed to the continuously bending LQ-curve, has been proposed as an alternative. Therefore, to compare the radiobiological impact of the different fractionation schemes, both the LQ and USC approaches were considered. Using the LQ model the surviving fraction *SF* in a fully oxygenated cell population following irradiation with dose *d* is given by:

(1a) $$\text{SF}=\exp\left(-\alpha \cdot d-\beta \cdot d^{2}\right)$$

where α and β are the radiosensitivity parameters for oxic conditions. The values of α and β used in all calculations were 0.33 Gy^-1^ and 0.038 Gy^-2^ respectively (α/β = 8.6 Gy), in accordance with the values reported by Park *et al.* (2008) for NSCLC.

If the universal survival curve model is applied, the corresponding expression for survival is given by:

(1b) $$\text{SF}=\left\{\begin{array}{ll} \exp\left(-\alpha \cdot d-\beta \cdot d^{2}\right) & \text{if}\;d\leq D_{T}\\ \exp\left[-\frac{1}{D_{0}}\cdot d+\frac{D_{q}}{D_{0}}\right] & \text{if}\;d\geq D_{T} \end{array}\right)$$

where *D*_q_ is the dose at which the tangent of the final slope *D*_0_ of the survival curve intercepts the horizontal axis at 100% survival and *D*_T_ is the threshold dose at which the LQ model transitions into the SHMT model. Values of *D*_0_, *D*_q_ and *D*_T_ for NSCLC were 1.25 Gy, 1.8 Gy and 6.2 Gy respectively [23].

As a result of the spatially varying oxygenation in the tumour, the radiosensitivity distribution will be non-homogeneous, causing a spatial variation in the effectiveness of the delivered dose. To account for the relative increase in the effect of ionizing radiation in the presence of oxygen [13,20], the survival models were modified to include oxygen modifying factors (OMFs) dependent on the local oxygen tension (*p*O_2_) [20]:

(2) $$\text{OMF}\left(pO_{2}\right)={\text{OMF}}_{\text{max}}\cdot \frac{k+pO_{2}}{k+{\text{OMF}}_{\text{max}}\cdot pO_{2}}$$

where *OMF*_max_ is the maximum relative resistance achieved in the absence of oxygen corresponding to an oxygen enhancement ratio (OER) of 3 and *k* is a reaction constant around 2.5-3 mmHg [25]. A value of *k* = 2.5 mmHg was used in the current simulations.

Thus, at voxel level, the surviving fraction of cells with a given sensitivity depending on the *p*O_2_ using the LQ model was calculated as [26]:

(3a) $$\text{SF}\left(d,pO_{2}\right)=\exp\left[-\frac{\alpha }{\text{OMF}\left(pO_{2}\right)}\cdot d-\frac{\beta }{{\text{OMF}}^{2}\left(pO_{2}\right)}\cdot d^{2}\right]\;$$

Similarly, the survival under various oxygenation conditions given by the universal survival curve model was calculated as:

(3b) $$\text{SF}\left(d,pO_{2}\right)=\left\{\begin{array}{ll} \exp\left[-\frac{\alpha }{\text{OMF}\left(pO_{2}\right)}\cdot d-\frac{\beta }{{\text{OMF}}^{2}\left(pO_{2}\right)}\cdot d^{2}\right] & \text{if}\;d\leq D_{T}\cdot \text{OMF}\left(pO_{2}\right)\\ \exp\left[-\frac{1}{D_{0}}\cdot \frac{d}{\text{OMF}\left(pO_{2}\right)}+\frac{D_{q}}{D_{0}}\right] & \text{if}\;d\geq D_{T}\cdot \text{OMF}\left(pO_{2}\right) \end{array}\right)$$

### Simulation of tumours

**Figure 1:**
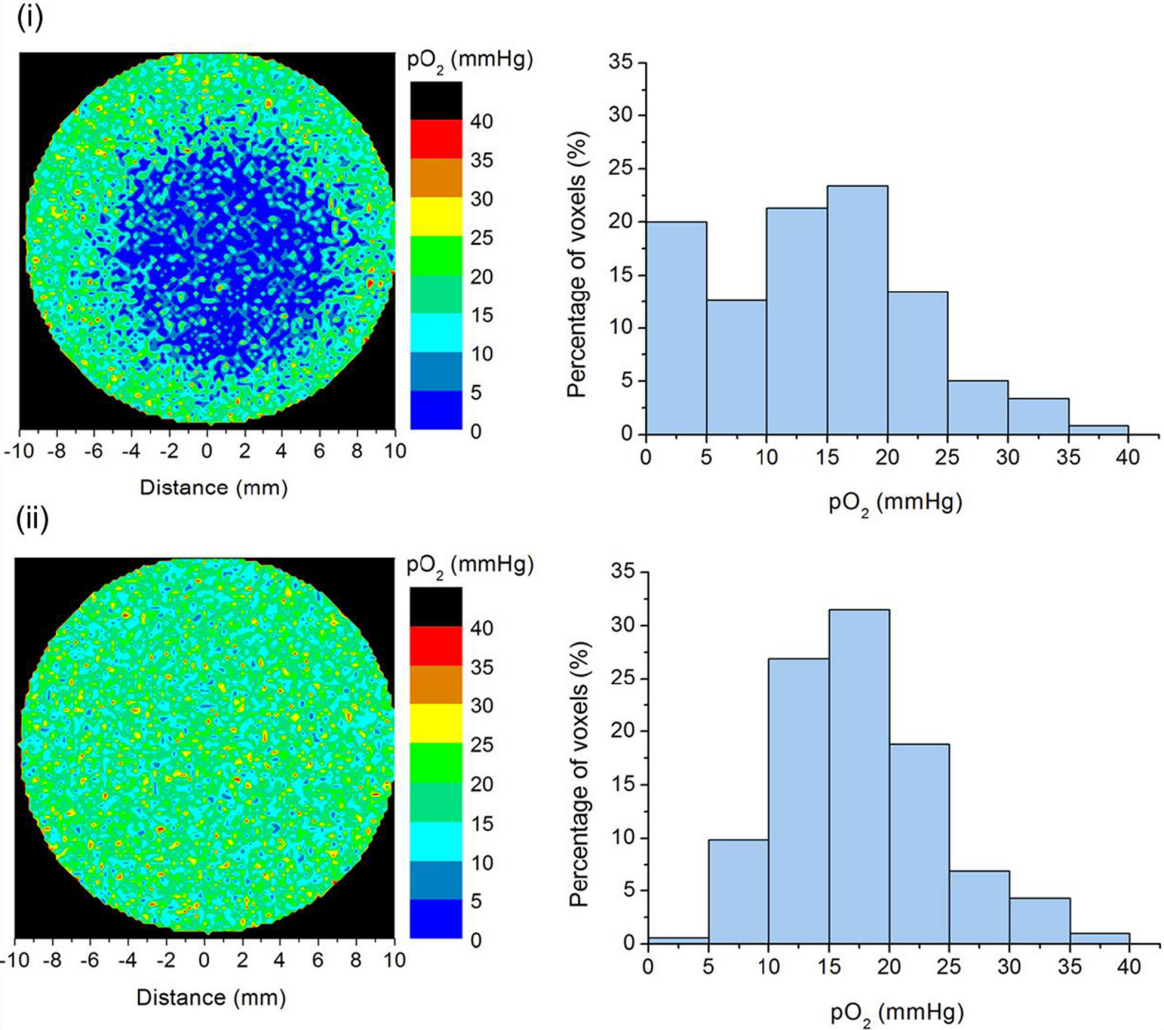
**Simulated tumours and their oxygenation.** Two-dimensional *p*O_2_-maps of cross-sections through the simulated tumours and the *p*O_2_-histograms of the oxygen tension values for the whole (3D) tumours. **i)** Hypoxic tumour with a 13 mm hypoxic core, overall *HF* ≈ 20%, core *HF* ≈ 64%, **ii)** Oxic tumour with an overall *HF* < 1%.

The three-dimensional tumour oxygenation was modelled based on biologically relevant inter-vessel distance (IVD) distributions derived from the experimental work by Konerding et al. (1999) [27]. By defining well-oxygenated and hypoxic regions and assigning IVD distributions with different average values to these regions, *in silico* tumours with different levels of hypoxic fraction (*HF*) were constructed based on the diffusion and consumption of oxygen. Two different oxygen distributions were considered for the 20 mm tumour in this study: a 13 mm hypoxic core resulting in an overall hypoxic fraction less than 5 mmHg (*HF*) of about 20% (corresponding to 64% hypoxia within the core) and an oxic tumour with less than 1% hypoxia heterogeneously distributed. Examples of cross-sections through these tumours and the corresponding oxygen partial pressure histograms are shown in Figure 1.

Acute hypoxia is associated with local changes in perfusion, which might take place between two consecutive SBRT fractions [16]. In order to investigate the impact of the resulting local oxygenation changes (LOC) on tumour control, the values of the oxygen tension were randomly locally redistributed at each fraction by randomly closing a fraction of the simulated vessels. For comparison, the case of static oxygenation was also investigated, by keeping the oxygen distribution the same in all fractions. No substantial improvement of overall tumour oxygenation associated with the slow reoxygenation of chronically hypoxic regions was simulated, because of the short overall treatment time in the SBRT treatment schedules considered for this study [13].

### Dose distribution and simulation of treatment

**Figure 2:**
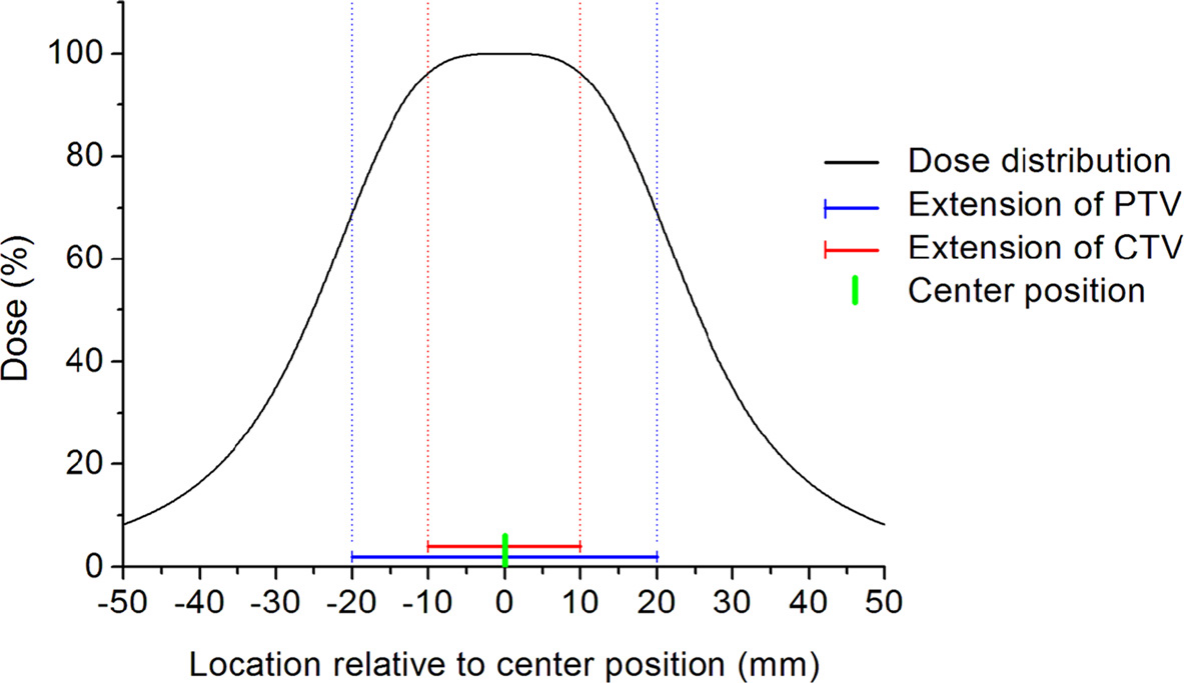
**Dose distribution for the simulation of SBRT treatments.** The clinically relevant dose distribution normalized to the maximum dose so that the percentage dose at the PTV periphery (20 mm from the centre) is 69% and the maximum dose is 100%. The extents of the CTV and PTV are marked with red and blue lines respectively.

For comparison with clinical data the irradiation of the modelled tumour with 20% overall hypoxic fraction (Figure 1i) was simulated using fractionated schedules currently employed in the clinic for the SBRT treatment of NSCLC [1-9] together with clinical prescription coverage of the planning target volume (PTV). The explicit number of fractions and the corresponding dose per fraction as well as the dose prescription planning details are given in Table 1. The diameter of the PTV was assumed to be 40 mm, corresponding to a clinical target volume (CTV) of 20 mm with an additional 10 mm margin. Using a clinically relevant dose distribution (Figure 2) the prescription details of the reported treatment schemes given in Table 1 were fulfilled in terms of dose escalation, dose to the PTV periphery and maximum dose.

**Table 1:** **SBRT fractionations for NSCLC with clinical outcome and calculated TCP for the 20% hypoxic tumour (Figure**1**i)**

**Reference**	**Dose scheme**	**Total dose (Gy)**	**Dose prescr.**	**Treatment outcome**		***TCP *** **for LQ (USC)**	
				**Overall survival**	**Local control**	**No LOC**	**LOC**
Hof *et al.* 2007 [3] (Germany)	26 Gy × 1	26	Isocenter	74.5% at 1 year^a^, 65.4% at 2 years^a^, 37.4% at 3 years^a^	100% at 1 year^b^, 72% at ≥ 2 years^b^	0% (0%)	N/A
Fritz *et al.* 2008 [4] (Germany)	30 Gy × 1	30	Isocenter	66% at 2 years, 53% at 3 years	81% at 3 years	0% (0%)	N/A
Zimmermann *et al.* 2005 [5](Germany 65% isodose)	12.5 Gy × 3	37.5	60%	80% at 1 year^c^, 75% at 2 years^c^	100% at 1 year^c^, 87% at 2 years^c^	7% (4%)	99% (98%)
Baumann *et al.* 2009 [6] (Sweden)	15 Gy × 3	45	67%	86% at 1 year, 65% at 2 years, 60% at 3 years	92% at 3 years	56% (44%)	100% (99%)
Olsen *et al.* 2011 [7] (USA, The Netherlands)	18 Gy × 3	54	80%	92/81% at 1 year^d^, 85/61% at 2 years^d^	99% at 1 year^d^, 91% at 2 years^d^	62% (50%)	100% (100%)
Haasbeek *et al.* 2010 [8] (The Netherlands)	20 Gy × 3	60	80%	85.7% at 1 year^e^, 54% at 2 years^e^, 45.1% at 3 years^e^	89% at 3 years^e^	96% (92%)	100% (100%)
Takeda *et al.* 2009 [9] (Canada)	10 Gy × 5	50	80%	90/63% at 3 years, (Stage 1A/1B)	93/96% at 3 years, (Stage 1A/1B)	0% (0%)	98% (98%)
Haasbeek *et al.* 2010 [8] (The Netherlands)	12 Gy × 5	60	80%	85.7% at 1 year^e^, 54% at 2 years^e^, 45.1% at 3 years^e^	89% at 3 years^e^	29% (28%)	100% (100%)
Haasbeek *et al.* 2010 [8] (The Netherlands)	7.5 Gy × 8	60	80%	85.7% at 1 year^e^, 54% at 2 years^e^, 45.1% at 3 years^e^	89% at 3 years^e^	0% (0%)	100% (100%)

^a^Based on doses 19–30 Gy to isocenter: 19 Gy (1 patient), 22 Gy (2), 24 Gy (7), **26 Gy (14)**, 28 Gy (10), 30 Gy (30).

^b^Patients receiving 26–30 Gy.

^c^Dose range 24–40 Gy, 69% was given 37.5 Gy, (2 patients had doses prescribed to 80% isodose).

^d^Doses prescribed to 60-90% isodose (median 84%), overall survival expressed as operable/inoperable patients and including other dose schemes.

^e^Based on 60 Gy total doses given in 3, 5 and 8 fractions: 3 × 20 Gy (33%), 5 × 12 Gy (50%), 8 × 7.5 Gy (17%).

N/A = Not Applicable.

In addition to the fractionation schemes clinically used, schedules in which the treatment is delivered in one, three or five fractions were employed for the modelled tumour types (Figure 1). For both cases, the clinically relevant dose distribution illustrated in Figure 2 was used with the fractional dose prescribed to the 69% isodose encompassing the PTV for the two tumour types considered. Prescription to the 69% isodose was chosen as representative of the current SBRT practice [1-9].

The response of the tumour was evaluated as the tumour control probability (*TCP*) calculated with a Poisson equation. Thus, the overall *TCP* was calculated as:

(4) $$\text{TCP}=\exp\left\{-\sum_{i=1}^{N_{\text{vox}}}N_{i}\Pi_{j=1}^{n}\text{SF}\left(d_{i},pO_{2\;i,j}\right)\right\}$$

where *n* is the number of fractions, *N*_vox_ is the total number of voxels, *N*_*i*_ is the number of cells in each voxel *i* and *d*_*i*_, *p*O_2 *i,j*_ and *SF*(*d*_*i*_, *p*O_2 *i,j*_), is the dose, the oxygen tension and the cell survival in voxel *i* at fraction *j*. By randomly re-distributing the *p*O_2_-values between voxels at each fraction, experimentally observed local variations in oxygenation between fractions [16] were simulated [19-21,28].

In the case of static oxygenation, when the oxygen tension of the individual voxels does not change between fractions, the surviving fraction of each voxel remains constant during the treatment and the equation above reduces to:

(5) $$\text{TCP}=\exp\left\{-\sum_{i=1}^{N_{\text{vox}}}N_{i}{\left[\text{SF}\left(d_{i},pO_{2\;i}\right)\right]}^{n}\right\}$$

In the current study the total number of clonogenic cells in the tumour was set to 10^8^.

The tumour control probability was determined for increasing values of total dose *D*, in order to generate typical dose–response curves by fitting a logit expression (6) to the resulting *TCP* data [29]:

(6) $$\text{TCP}=100\cdot \frac{1}{1+{\left(\frac{D_{50}}{D}\right)}^{4\cdot \gamma }}$$

*D*_50_ is the total dose required for a tumour control probability of 50% and γ is the slope of the curve at 50% *TCP*, similar to the clinical fit of dose–response curves.

## Results

The TCP values for the 20% hypoxic tumour (Figure 1i) calculated using the clinical dose prescription schemes are presented in Table 1 together with the reported clinical outcome. For single-fraction schedules, there is a large difference between the predicted *TCP* and the clinically observed values of local control. For multifraction schemes, a trend of better agreement between clinical outcome and simulations assuming LOC compared to the case assuming static oxygenation was observed. For most of the schedules the choice of survival model between LQ and USC seems to have little, if any, impact on the outcome in terms of *TCP*.

**Figure 3:**
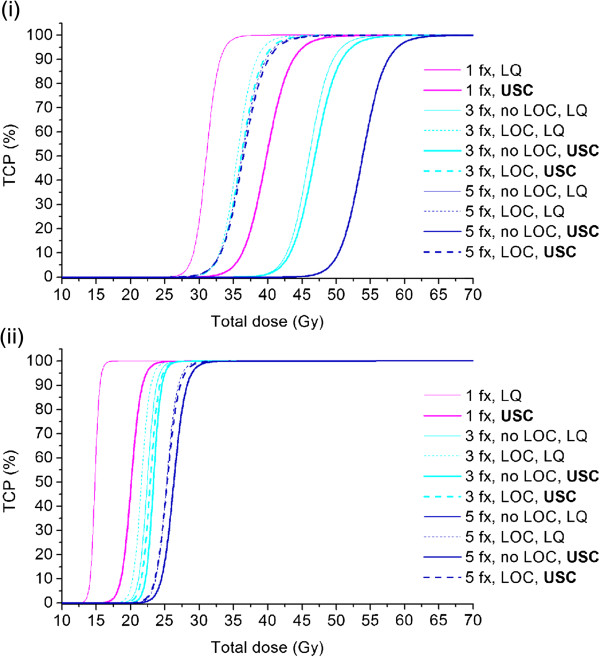
***TCP *curves using the LQ and USC models.** *TCP* curves for a tumour with **i)** 20% overall hypoxic fraction located centrally and **ii)** 1% hypoxic fraction, heterogeneously distributed, with and without inter-fraction LOC calculated with the linear-quadratic model and the universal survival curve as a function of total dose prescribed to the PTV-encompassing 69% isodose.

Figure 3i and ii show the *TCP* curves obtained for the clinically-relevant theoretical schedules of different fractionations when either the linear-quadratic or the universal survival curve model was used to calculate the surviving fraction in the hypoxic (*HF* ≈ 20%) and the oxic (*HF* < 1%) tumours (Figures 1i and ii). In Table 2 summarizing the *D*_50_ values, it can be observed that the *D*_50_ increases as the number of fractions is increased. However, the increase is not as large as might have been expected from performing a simple calculation of the corresponding equivalent isoeffective dose using a typical biological effective dose (BED) conversion [22,30]. The impact of increasing the number of fractions is more pronounced for static oxygenation. For example in Figure 3i, the curves representing 1, 3 and 5 fractions (labelled "no LOC, LQ") lead to *D*_50_ values of 31.0, 46.0 and 53.9 Gy respectively. Assuming LOC, the 7.9 Gy difference in *D*_50_ between 3 and 5 fractions is reduced to only 0.6 Gy (35.6 Gy vs. 36.2 Gy for 3 and 5 fractions respectively). Furthermore, the difference in *D*_50_ between one and five fractions is only 5.2 Gy, (*D*_50_ = 31.0 Gy for one fraction and 36.2 Gy for five fractions assuming LOC).

**Table 2:** **Values of *D***_**50 **_**for the clinically-relevant theoretical fractionation schedules**

**Hypoxic fraction**	**Survival model**	***D***_**50 **_**(Gy)**					
		**1 fraction**		**3 fractions**		**5 fractions**	
		No LOC	LOC	No LOC	LOC	No LOC	LOC
< 1%	LQ	14.8	N/A	22.4	21.6	26.3	25.3
	USC	20.1	N/A	23.3	22.9	26.3	25.3
≈ 20%	LQ	31.0	N/A	46.0	35.6	53.9	36.2
	USC	39.8	N/A	46.9	36.1	53.8	36.4

N/A = Not Applicable.

A certain level of *TCP* requires higher doses with the USC than with the LQ model, in accordance with the predicted over-estimation of clonogenic cell killing by the LQ model. For single fraction doses, the difference between the LQ- and USC-curves is quite large. For three and five fractions however, the difference between using either of the two models seems to be negligible, the two curves for 5 fractions being visually indistinguishable for both tumour types assuming LOC. Noticeable in Figure 3i and ii is the smaller range of *D*_50_ for the *TCP* curves obtained with USC model (thick curves) compared to the curves obtained with the LQ model (thin curves). This reflects the lower fractionation-sensitivity of the USC model at high fractional doses [31].

An interesting feature of the curves in Figure 3i is that for a given level of *TCP*, the single-fraction curve obtained with the USC model predicts a higher total dose than some of the curves representing three and five fractions. Although this might seem counterintuitive, it is a consequence of the way the universal survival curve model is constructed. At doses per fraction above the transition dose *D*_T_, the universal survival curve predicts a difference in survival compared to the LQ model that increases with increasing dose. For the high doses required in single-fraction treatments, the difference is most pronounced, as reflected by the large difference in *D*_50_ between the single-fraction *TCP*-curves obtained with the LQ and USC models in Figure 3i. For a total dose delivered in three or five fractions, the fractional dose is much closer to the transition dose and the predicted survival is thus higher.

## Discussion

Hypoxia is a common feature of solid tumours that is considered responsible for the failure of many treatment approaches [32]. It has been shown to affect non-small cell lung cancer (NSCLC) tumours, where more than 80% of the investigated patients had a fractional hypoxic volume (FHV) over 20% and the median FHV was 47.6% [33].

Reoxygenation of tumours is thought to be an effective way to increase local control and it represents one of the radiobiological rationales for conventionally-fractionated radiotherapy. However, the impact of inter-fraction local oxygenation changes for extremely hypofractionated SBRT employing high doses per fraction has not been fully investigated. SBRT treatments are usually very short and therefore they do not allow enough time for the global reoxygenation that results from tumour shrinkage in longer treatments [13]. Fluctuations of acute hypoxia on the microscale, as described by Ljungkvist *et al.*[16] thus remain the main mechanism that could change tumour oxygenation for extremely fractionated schedules. The outcome of the present study suggests that inter-fraction LOC is a process that could strongly modify the response of hypoxic tumours, possibly explaining the current success of SBRT in treating hypoxic tumours.

The results for the clinical multifraction schedules presented in Table 1 indicate a trend of better agreement between local control and the calculated *TCP* values assuming LOC, as opposed to the case of static oxygenation. This could indicate that local oxygenation changes might take place between fractions in clinical SBRT patients. As no information of the oxygenation or number of clonogenic cells is available for the tumours included in the clinical studies, a direct comparison between calculated values and the reported local control is difficult to make. For the single fraction schemes, the difference between the observed local control and the calculated values of *TCP* is large. This could be explained by the limited knowledge of tumour response to the high doses delivered in single-fraction treatments. It has been hypothesized that there might be processes leading to increased cell death only taking place at these high doses such as vascular damage [34]. As such effects are not included in the present modelling, they could be one of the reasons for the observed discrepancies.

The dose–response curves and corresponding values of *D*_50_ (Table 2) show that a great decrease in dose per fraction can be expected if LOC is assumed, the total doses for three and five fractions being almost equally low. This offers an interesting point of view for the issue of fractionation for stereotactic treatments. While extremely hypofractionated schedules may not be preferred from the point of view of the conventional fractionations where the focus is on the differential between tumour response and normal tissue damage, they might provide an advantage for stereotactic treatments that are based on the limiting of the ‘red shell’, the high-risk zone of normal tissues receiving therapeutic doses [35]. Thus, shorter schedules might appeal both to patients that would have to go through fewer treatment sessions and for the radiotherapy departments as they will free valuable accelerator time that could in turn be used to increase patient throughput. Nevertheless, this would apply only if LOC take place during the treatment, as otherwise much higher doses would be needed to achieve the same control rates.

The simulations in the present study have been performed using both the LQ model and the universal survival curve (USC) model which is an empirical extension of the LQ model. The suitability of the LQ model for high doses has been intensely debated in recent years [24] focusing on the possibility of overestimating cell kill at high doses like those used in SBRT. This has led to the development of the universal survival curve model which is thought to better fit the experimental data in the high-dose range [23], although the mathematical framework and the lack of mechanistic basis of the model has been criticized [36]. The results of the current work indicate that for multifraction schedules the difference in terms of the calculated *TCP* between using either the LQ or USC formalism is not considerable.

The present study adds to the results of two previous studies based on the LQ formalism [17,18]. Indeed, comparing the results in Figure 3i and ii and Table 2, it can be seen that tumours with increased hypoxia would require higher radiation doses to achieve a high *TCP*, especially in the absence of inter-fraction LOC, which is in agreement with the proposal of Carlson and colleagues [18]. Ruggieri and colleagues argued that intra-tumour simultaneous dose-boosting is capable to counteract hypoxic radioresistance [17]. This statement is not in contradiction with the results of Carlson *et al.* or with the results of the present simulations. Indeed, dose escalations towards the centre of the tumour will increase cell kill and therefore lead to better tumour control compared to homogeneous dose distributions, especially if the hypoxic areas are centrally located. However for non-gated treatments, the tumour movement relative to the treatment beams could bring the tumour towards the lower dose regions at the margin of the PTV, which could in turn lead to a shift of the dose response curve to higher doses. Nevertheless, choosing suitable PTV-to-CTV margins might minimize the impact of this factor and the expected differences will be small.

## Conclusion

The results of this study illustrate the interplay that could be expected between total dose, fractionation, hypoxia, and the dynamics of oxygenation for SBRT treatments. They suggest that extreme hypofractionation, as low as one single dose fraction, should be pursued with caution so that the current success of SBRT should not be jeopardized.

## Competing interests

The authors’ declare that they have no competing interest.

## Authors’ contributions

All authors have been involved in the study and the manuscript. EL participated in the design of the study, the theoretical modelling and simulations, the analysis and interpretation of data and drafted the manuscript, LA participated in the analysis and interpretation of data and reviewing the manuscript, AD provided the basic software for the theoretical simulations, IL participated in the design of the study, interpretation of data and reviewing the manuscript, PW participated in the interpretation of data and reviewing the manuscript and IT-D coordinated the design of the study, analysis and interpretation of data. All authors read and approved the manuscript.

## References

[B1] Lax I Blomgren H Näslund I Svanström R Stereotactic radiotherapy ofmalignancies in the abdomen Acta Oncol 1994 33 677 683 10.3109/02841869409121782 7946448

[B2] Blomgren H Lax I Näslund I Svanström R Stereotactic high dose fraction radiation therapy of extracranial tumours using an accelerator Acta Oncol 1995 34 861 870 10.3109/02841869509127197 7576756

[B3] Hof H Muenter M Oetzel D Hoess A Debus J Herfarth K Stereotactic single-dose radiotherapy (radiosurgery) of early-stage nonsmall-cell lung cancer (NSCLC) Cancer 2007 110 148 155 10.1002/cncr.22763 17516437

[B4] Fritz P Kraus HJ Blaschke T Mühlnickel W Strauch K Engel-Riedel W Chemaissani A Stoelben E Stereotactic, high single-dose irradiation of stage I non-small cell lung cancer (NSCLC) using four-dimensional CT scans for treatment planning Lung Cancer 2008 60 193 199 10.1016/j.lungcan.2007.10.005 18045732

[B5] Zimmermann FB Geinitz H Schill S Grosu A Schratzenstaller U Molls M Jeremic B Stereotactic hypofractionated radiation therapy for stage I non-small cell lung cancer Lung Cancer 2005 48 107 114 10.1016/j.lungcan.2004.10.015 15777977

[B6] Baumann P Nyman J Hoyer M Wennberg B Gagliardi G Lax I Drugge N Ekberg L Friesland S Johansson KA Lund JÅ Morhed E Nilsson K Levin N Paludan M Sederholm C Traberg A Wittgren L Lewensohn R Outcome in a prospective phase II trial of medically inoperable stage I non-small-cell lung cancer patients treated with stereotactic body radiotherapy J Clin Oncol 2009 27 3290 3296 10.1200/JCO.2008.21.5681 19414667

[B7] Olsen JR Robinson CG El Naqa I Creach KM Drzymala RE Bloch C Parikh PJ Bradley JD Dose–response for stereotactic body radiotherapy in early-stage non-small-cell lung cancer Int J Radiat Oncol Biol Phys 2011 81 e299 e303 10.1016/j.ijrobp.2011.01.038 21477948

[B8] Haasbeek CJA Lagerwaard FJ Antonisse ME Slotman BJ Senan S Stage I nonsmall cell lung cancer in patients aged ≥ 75 years: outcomes after stereotactic radiotherapy Cancer 2010 116 406 414 10.1002/cncr.24759 19950125

[B9] Takeda A Sanuki N Kunieda E Ohashi T Oku Y Takeda T Shigematsu N Kubo A Stereotactic body radiotherapy for primary lung cancer at a dose of 50 Gy total in five fractions to the periphery of the planning target volume calculated using a superposition algorithm Int J Radiat Oncol Biol Phys 2009 73 442 448 10.1016/j.ijrobp.2008.04.043 18990507

[B10] Song CW Park H Griffin RJ Levitt SH Levitt SH, Purdy JA, Perez CA, Poortmans P Radiobiology of stereotactic radiosurgery and stereotactic body radiation therapy Technical Basis of Radiation Therapy – Practical Clinical Applications 2012 5 Berlin, Heidelberg: Springer-Verlag 51 61

[B11] Withers HR Taylor JMG Maciejewski B The hazard of accelerated tumour clonogen repopulation during radiotherapy Acta Oncol 1988 27 131 146 10.3109/02841868809090333 3390344

[B12] Paganetti H Changes in tumour cell response due to prolonged dose delivery times in fractionated radiation therapy Int J Radiat Oncol Biol Phys 2005 63 892 900 10.1016/j.ijrobp.2005.07.953 16199319

[B13] Hall EJ Giaccia AJ Radiobiology for the Radiologist 2006 6 Philadelphia: Lippincott/ Williams & Wilkins 85–89 378–379

[B14] Kallman RF The phenomenon of reoxygenation and its implications for fractionated radiotherapy Radiology 1972 105 135 142 10.1148/105.1.135 4506641

[B15] Brown JM Evidence for acutely hypoxic cells in mouse tumours, and a possible mechanism of reoxyenation Br J Radiol 1979 52 650 656 10.1259/0007-1285-52-620-650 486895

[B16] Ljungkvist AS Bussink J Kaanders JH Wiedenmann NE Vlasman R van der Kogel AJ Dynamics of hypoxia, proliferation and apoptosis after irradiation in a murine tumour model Rad Res 2006 165 326 336 10.1667/RR3515.1 16494521

[B17] Ruggieri R Naccarato S Nahum AE Severe hypofractionation: non-homogeneous tumour dose delivery can counteract tumour hypoxia Acta Oncol 2010 49 1304 1314 10.3109/0284186X.2010.486796 20500031

[B18] Carlson DJ Keall PJ Loo BW Jr Chen ZJ Brown JM Hypofractionation results in reduced tumour cell kill compared to conventional fractionation for tumours with regions of hypoxia Int J Radiat Oncol Biol Phys 2011 79 1188 1195 10.1016/j.ijrobp.2010.10.007 21183291 PMC3053128

[B19] Dasu A Toma-Dasu I Karlsson M Theoretical simulation of tumour oxygenation and results from acute and chronic hypoxia Phys Med Biol 2003 48 2829 2842 10.1088/0031-9155/48/17/307 14516104

[B20] Dasu A Toma-Dasu I Karlsson M The effects of hypoxia on the theoretical modelling of tumour control probability Acta Oncol 2005 44 563 571 10.1080/02841860500244435 16165915

[B21] Toma-Dasu I Dasu A Brahme A Dose prescription and optimisation based on tumour hypoxia Acta Oncol 2009 48 1181 1192 10.3109/02841860903188643 19863227

[B22] Barendsen GW Dose fractionation, dose rate and iso-effect relationships for normal tissue responses Int J Radiat Oncol Biol Phys 1982 8 1981 1997 10.1016/0360-3016(82)90459-X 6759484

[B23] Park C Papiez L Zhang S Story M Timmerman RD Universal survival curve and single fraction equivalent dose: useful tools in understanding potency of ablative radiotherapy Int J Radiat Oncol Biol Phys 2008 70 847 852 10.1016/j.ijrobp.2007.10.059 18262098

[B24] Kirkpatrick JP Brenner DJ Orton CG Point/Counterpoint. The linear-quadratic model is inappropriate to model high dose per fraction effects in radiosurgery Med Phys 2009 36 3381 3384 10.1118/1.3157095 19746770

[B25] Alper T Cellular radiobiology 1979 Cambridge, UK: Cambridge University Press

[B26] Toma-Dasu I Dasu A Modelling tumour oxygenation, reoxygenation and implications on treatment outcome Comput Math Methods Med 2013 2013 141087 23401721 10.1155/2013/141087 PMC3557613

[B27] Konerding MA Malkusch W Klapthor B Evidence for characteristic vascular patterns in solid tumours: quantitative studies using corrosion casts Br J Cancer 1999 80 724 732 10.1038/sj.bjc.6690416 10360650 PMC2362271

[B28] Antonovic L Lindblom E Dasu A Bassler N Furusawa Y Toma-Dasu I Clinical oxygen enhancement ratio of tumors in carbon ion radiotherapy: The influence of local oxygenation changes J Radiat Res 2014 Epub ahead of print, doi:10.1093/jrr/rru020 10.1093/jrr/rru020 PMC4240637 24728013

[B29] Dasu A Toma-Dasu I Prostate alpha/beta revisited – an analysis of clinical results from 14 168 patients Acta Oncol 2012 51 963 974 10.3109/0284186X.2012.719635 22966812

[B30] Dale RG The application of the linear-quadratic dose-effect equation to fractionated and protracted radiotherapy Br J Radiol 1985 58 515 528 10.1259/0007-1285-58-690-515 4063711

[B31] Wennberg B Lax I The impact of fractionation in SBRT: analysis with the linear quadratic model and the universal survival curve model Acta Oncol 2013 52 902 909 10.3109/0284186X.2012.728292 23327339

[B32] Overgaard J Hypoxic radiosensitization: adored and ignored J Clin Oncol 2007 25 4066 4074 10.1200/JCO.2007.12.7878 17827455

[B33] Rasey JS Koh WJ Evans ML Peterson LM Lewellen TK Graham MM Krohn KA Quantifying regional hypoxia in human tumors with positron emission tomography of [18 F]Fluoromisonidazole: a pretherapy study of 37 patients Int J Radiat Oncol Biol Phys 1996 36 417 428 10.1016/S0360-3016(96)00325-2 8892467

[B34] Park HJ Griffin RJ Hui S Levitt SH Song CW Radiation-induced vascular damage in tumors: implications of vascular damage in ablative hypofractionated radiotherapy (SBRT and SRS) Rad Res 2012 177 311 327 10.1667/RR2773.1 22229487

[B35] Yang J Fowler JF Lamond JP Lanciano R Feng J Brady LW Red shell: defining a high-risk zone of normal tissue damage in stereotactic body radiation therapy Int J Radiat Oncol Biol Phys 2010 77 903 909 10.1016/j.ijrobp.2009.12.069 20400240

[B36] Tomé WA Universal survival curve and single fraction equivalent dose: useful tools in understanding potency of ablative radiotherapy: in regard to Parks et al. (Int J Radiat Oncol Biol Phys 2008;72:1620–1621) Int J Radiat Oncol Biol Phys 2009 73 1286 19251106 10.1016/j.ijrobp.2008.12.001

